# Challenging Visualization of Sentinel Lymph Nodes in Upper Urinary Tract Urothelial Carcinoma

**DOI:** 10.3390/jcm10235465

**Published:** 2021-11-23

**Authors:** Wojciech Polom, Wojciech Cytawa, Anna Polom, Mikołaj Frankiewicz, Edyta Szurowska, Piotr Lass, Marcin Matuszewski

**Affiliations:** 1 Urology Clinic, Medical University of Gdansk, 80-210 Gdansk, Poland; mfrankiewicz@gumed.edu.pl (M.F.); matmar@gumed.edu.pl (M.M.); 2 Nuclear Medicine Department, Medical University of Gdansk, 80-210 Gdansk, Poland; wojciech.cytawa@gumed.edu.pl (W.C.); piotr.lass@gumed.edu.pl (P.L.); 3 Radiology Department, Medical University of Gdansk, 80-210 Gdansk, Poland; anka.polom@gumed.edu.pl (A.P.); edyta.szurowska@gumed.edu.pl (E.S.)

**Keywords:** upper urinary tract tumor, sentinel lymph node, lymphangiography, ureterorenoscopy, single-photon emission-computed tomography/computed tomography

## Abstract

Purpose: The purpose of this study was to assess the possibility of detecting sentinel lymph nodes (SLNs) and to perform analysis of lymphatic outflow in patients with suspicion of upper tract urothelial carcinoma (UTUC) with the use of a radioisotope-based technique. Methods: During 2018–2021, a prospective study was conducted on 19 patients with the suspicion of UTUC and for whom diagnostic ureterorenoscopy (URS) was planned. Technetium-99m (^99m^Tc) nanocolloid radioactive tracer injection and a tumor biopsy were performed for staging procedures. Three-dimensional (3D) reconstruction and fusion of images were performed for better localization of lymph nodes (LNs). Detection of SLNs and the analysis of the radiotracer outflow was conducted with the use of single-photon emission-computed tomography/computed tomography (SPECT/CT) lymphangiography. Results: The mean age of the patients was 73.4 years; 7 (36%) were male. Pathological staging from the biopsy was T0—8 (42%), Ta—7 (36%), T1—4 (21%). SLNs were detected in two of 19 cases (10%). In one patient a single SLN (5.3%) was visualized, and in another case (5.3%), multiple (double) radioactive lymph nodes were visualized. In 17 out of the 19 (89.5%) cases, no lymphatic outflow was observed, and out of these five cases (26.3%) of gravitational leakage of injected radiotracer to the retroperitoneal space was noted. Conclusions: We demonstrated that detection of SLNs in the upper urinary tract is possible yet challenging. Radiotracer injection in the upper urinary tract during ureterorenoscopy is difficult to perform, and the expected result of injection is unsatisfactory. Lymphatic outflow from the tumor site to the first LNs in our studied group of patients is visible in 10.5% of cases. SPECT/CT lymphangiography in cases of UTUC may provide valuable information about a patient’s individual anatomy of the lymphatic system and the position of the first lymph nodes draining lymph with potential metastatic cells from the tumor.

## 1. Introduction

UTUC is an uncommon urinary tract malignancy. It accounts for 5–10% of urothelial carcinomas (UCs) [1,2]. Most UCs are localized in the bladder site, where they represent the most common urinary tract malignancies [3]. Multifocal tumors can be diagnosed in 10–20% of cases [4]. Patients with ureteral and/or multifocal tumors have worse prognoses than those diagnosed with renal pelvic tumors [5,6]. What is more, carcinoma in the upper urinary tract is present as a concomitant malignancy in 11–36% of cases [4], and concurrent bladder cancer is present in 17% of cases [7]. Recurrence in the bladder localization occurs in 22–47% of patients after UTUC treatment [8]. Moreover, contralateral upper tract localization is the site of recurrence in 2–6% of patients [9]. UTUC is classified as an aggressive cancer. In 60% of cases invasive cancer is diagnosed [10] with 7% probability of presence of metastatic disease [4]. This cancer is three times more common in men than in women, and it concerns mostly elderly populations, with peak incidence in patients aged 70–90 years [11]. Hereditary UTUCs are associated with hereditary nonpolyposis colorectal carcinoma (HNPCC) [12], although there are several environmental risk factors responsible for the development of UTUC [13]. Among them we can distinguish two factors with strong evidence of affecting the development of the disease: smoking, which increases the risk of UTUC from 2.5 to 7.0 times [14,15], and aristolochic acid. Moreover, alcohol consumption is an independent risk factor for UTUC development, in which a dose-response relationship was observed [16]. 

The histology of upper urinary tract carcinoma varies. Pure non-urothelial histology is very rare [17,18], but in 25% of cases variants are present [19,20]. Squamous cell carcinoma can develop based on inflammatory diseases and infections caused by urolithiasis [21,22]. Rare variants include sarcomatoid and urothelial carcinoma with inverted growth [23]. 

Lymphatic drainage and the anatomic positions of lymph nodes of the upper urinary tract are not yet clearly identified. The complexity of the lymphatic system and the anatomical differences between the right side and the left side of the abdominal cavity cause problems in accurate identification of lymph nodes and the performance of lymphadenectomy. This finding was described by the authors performing SLNB in cases of renal cancer, where they have found this technique useful for pre- and intraoperative SLN identification [24]. The concept of SLNB is based on the theory that finding and examining the first lymph node draining the lymph from the tumor site provides information about the status of the regional lymphatic system. It was proven that the lymphadenectomy template has a greater impact on patient survival than the number of lymph nodes removed during surgery [25]. In cases of TaT1, UTUC lymph-node dissection is infrequent, and it seems to be unnecessary because lymph node retrieval reports only 2.2% of T1 vs. 16% of pT2-4 tumors [26,27]. The probability of lymph node-positive disease is related to pT classification [28]. In most cases, lymphadenectomy is based on an anatomical template-based approach [29], although it is still not possible to standardize indication or the extent of lymph nodes dissection (LND). It is accepted that if indicated, lymphadenectomy should be performed on the side of the affected ureter as follows: retroperitoneal LND for higher ureteral tumors and/or tumors of the renal pelvis (right side: border of the vena cava or right side of the aorta; left side: border of the aorta) [26,30,31]. In our study we assess the possibility of detecting SLNs in the upper urinary tract and perform an analysis of the lymphatic outflow in patients with the suspicion of UTUC with the use of a radioisotope-based technique. 

## 2. Materials and Methods

Between March 2018 and January 2021, a prospective study was conducted on 19 patients with suspected tumor mass of the upper urinary tract diagnosed preoperatively in computed tomography urography (CTU). The study was approved by the local ethics committee under the number NKBBN/522/2017-2018. All procedures performed in this study involving human participants were in accordance with the ethical standards as laid out in the 1964 Declaration of Helsinki and its later amendments. Informed consent was obtained from all individual participants included in the study. Patients who had been previously exposed to factors that could disrupt lymphatic drainage, such as previous radiotherapy or chemotherapy, and patients after previous surgical treatment in the abdomen or pelvis were excluded from the study. Table 1 presets detailed information about studied group of patients.

In each patient the following procedures were performed: diagnostic endoscopy of the bladder, ureter and collecting system evaluation, tumor localization and biopsy of the suspected tumor mass, and urine cytology to diagnose potential concomitant carcinoma in situ.

**Figure 1:**
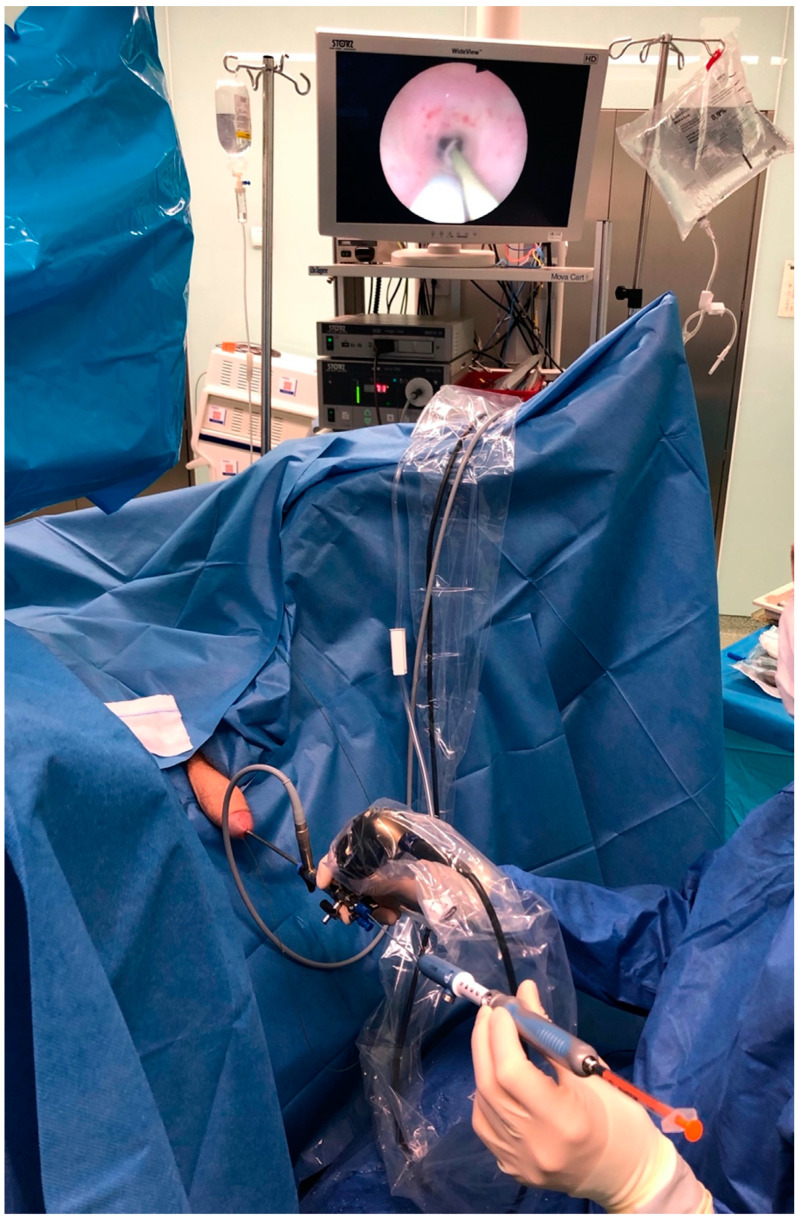
The use of a flexible Echotip Ultra endoscopic needle (Cook) during ureterorenoscopic radiotracer injection.

Injection of technetium-99m (^99m^Tc) labelled human serum albumin nano-sized colloid particles (^99m^Tc-HSA, Nano-scan^®^, 37 MBq per 1 mL 0.9% NaCl, diameter of particles 10 to 100 nm, IDB Holland Advanced Accelerator Applications, Baarle-Nassau, The Netherlands) was performed during diagnostic ureterorenoscopy under general anesthesia. In each case, a semirigid single-channel Olympus ureterorenoscope with an outer diameter of 6.4 to 8.6 Fr was used for the endoscopic procedure. Injection was performed with the use of a flexible Echotip Ultra endoscopic needle (Cook, Spencer, IN, USA) with adjustable needle length for the submucosal injection (Figure 1). 

We have divided the urinary tract into three parts where the injections were performed according to the suspected tumor localization—the distal ureter, the middle ureter and the upper ureter also containing the renal pelvis and calyxes of the kidney. 

**Figure 2:**
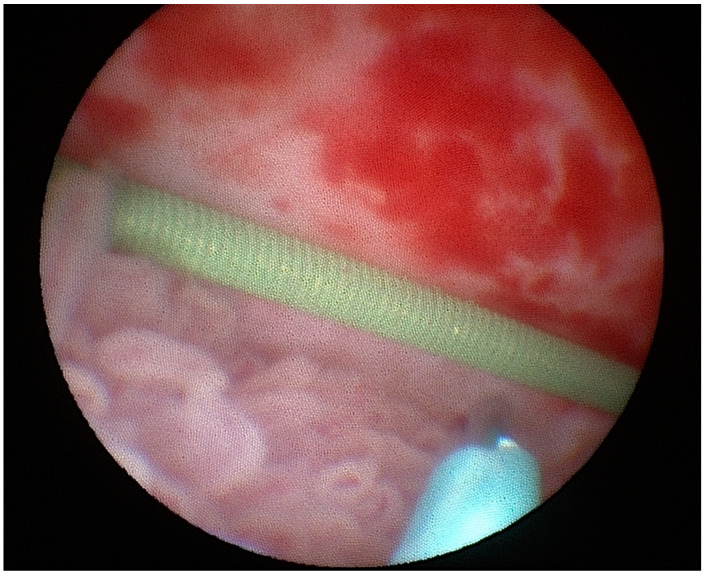
Injection of the radiotracer in the case of a tumor localized in collecting system of the kidney.

The mucosa and muscle layer of the ureter is very thin as compared to the bladder or gastrointestinal tract, so the length of the needle was adjusted to 2 mm and injection of the radiotracer was performed very superficially under the mucosa into the healthy urothelium around the tumor, and it was divided into four portions of 9.25 MBq per injection (Figure 2). Hybrid SPECT/CT lymphangiography was performed 24 ± 3 h after the radiotracer injection in each patient using a Symbia T6 SPECT/CT (Siemens, Erlangen, Germany) dual-head γ-camera equipped with a 6-row, spiral CT scanner. The SPECT/CT acquisition parameters were as follows: 128 × 128 matrix, 64 frames at 30 s each, low dose CT without intravenous contrast media. In every case reconstruction and image fusion were performed with Syngo software (Copyright© Siemens AG, Berlin and Munich 2008). Moreover, 3D image reconstruction of superimposed images was created for better localization of SLNs. Any focal activity of radiotracers detected in the local lymphatic outflow region, apart from the site of injection and spillage, was considered an SLN.

## 3. Results

### 3.1. Patients’ Characteristics

The mean age of the patients was 73.4 years; 7 (36%) were male. Pathological staging from the biopsy was T0–8 (42%), Ta–7 (36%), T1–4 (21%). Low-grade tumor was diagnosed in the majority of patients–7 (36%), and high-grade in four cases (21%). In four patients (21%) concomitant bladder cancer was diagnosed. T0 patients from the biopsy were not excluded from the group because it did not change the assessment of the radiotracer outflow from the upper urinary tract in tumor-suspected patients.

### 3.2. Imaging of Lymphatic Outflow

**Figure 3:**
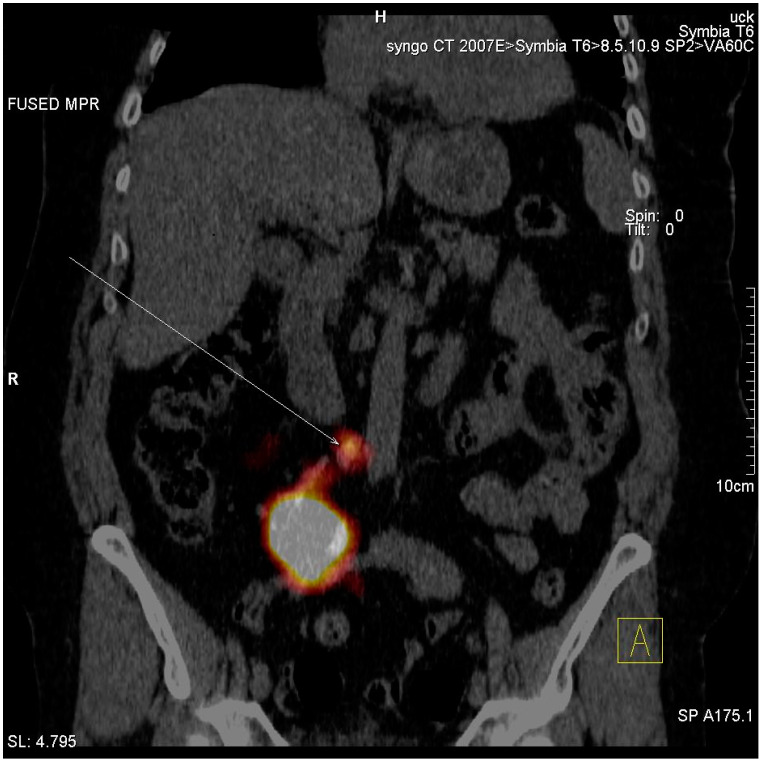
SPECT/CT example of lymphatic outflow from the right renal pelvis caudally and medially to the paraaortic region.

**Figure 4:**
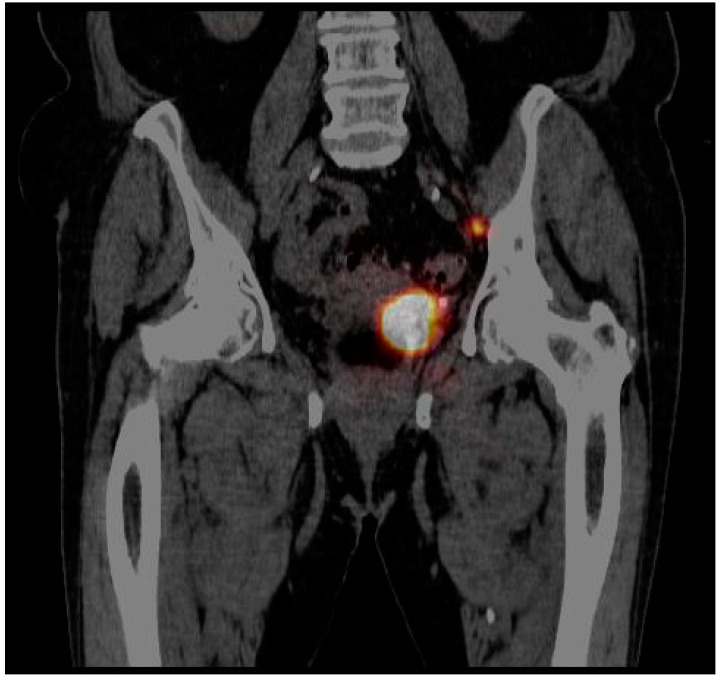
SPECT/CT example of lymphatic outflow from the left distal ureter cranially and laterally to the left outer iliac vein region.

**Figure 5:**
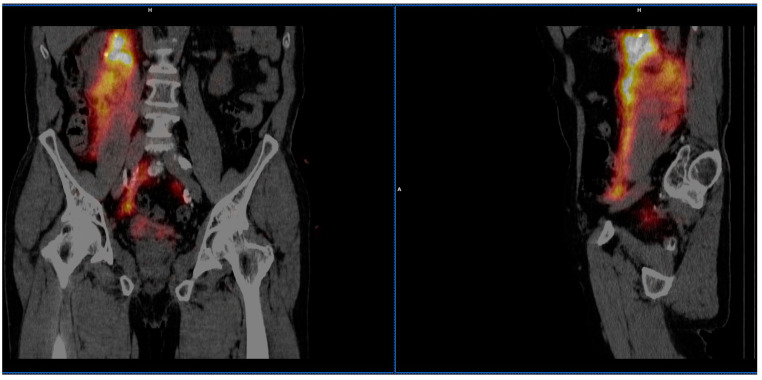
SPECT/CT example of no lymphatic outflow and radiotracer spillage after radiotracer injection performed at the site of the tumor located in the renal pelvis.

SLNs were detected in two of 19 cases (10.5%). In the first patient where the tumor was localized in the right renal pelvis the lymphatic outflow was directed medially and caudally from the injection site, resulting in the appearance of a single, unilateral SLN in the paraaortic region just above the bifurcation of the aorta (Figure 3). In the other patient, with the tumor located in the distal part of the left ureter, the lymphatic drainage was headed laterally and cranially from the injection site, visualizing two unilateral SLNs—one with clear focal uptake behind the left external iliac artery just below the division of the common iliac artery, and the other with faint focal uptake located slightly higher (Figure 4). In the remaining 17 cases (89.5%), no clear SLNs were observed, although in five cases (26.3%) SPECT/CT imaging revealed diffused outflow of radiotracer from the injection site caudally, through the retroperitoneal space, outside the urinary tract, along the ipsilateral iliopsoas muscle (Figure 5). No crossover phenomena, i.e., lymphatic outflow to the contralateral site, were observed. 

## 4. Discussion

The prognostic and predictive value of SLN status has been proved in many human malignancies (X-Y). “Positive” SLN (containing metastatic tumor cells) correlates with poorer prognosis for the patient. It also justifies decisions about regional lymphadenectomy and may indicate adjuvant oncological treatment. 

There are several studies regarding lymphatic outflow assessment and SLNB in urological malignancies such as penile cancer [32], prostate cancer, testicular cancer, and bladder cancer. Sherif et al. detected SLNs in 85% (11 of 13) of patients with bladder cancer after intravesical injection of radiotracer followed by lymphoscintigraphy for lymphatic drainage visualization and SLN detection [33]. A previous study of ours demonstrated that preoperative detection of SLNs in cases of bladder cancer with the use of SPECT/CT and intraoperative γ-ray detection probe of those SLNs gives similar results [34]. 

Proper staging has an important role in predicting patient outcome in various urological tumors, including UTUC. Lymphatic tumor spread is a predictor of worse outcome. It is difficult to evaluate the overall incidence of lymph node metastases in UTUCs, since lymphadenectomy is not performed routinely during nephroureterectomy procedures. In cases of this malignancy, pNx status varies between 25% to 86%, according to different authors [35,36]. Nevertheless, metastatic LNs are reported in 10 to 40% of cases, and they are independent of tumor localization (renal pelvis or ureter) [5,37]. pN stage was found to be an independent predictor of cancer-specific mortality, which was described by Lughezzani et al. [38]. Their observation was confirmed by other authors [10,25,26,27,28,29,30,31,32,33,34,35,36,37,38,39,40,41]. Roscigno et al. proved that pNx status is associated with worse prognosis than pN0 in cases of pT2-4 tumors, and they recommended lymphadenectomy to improve staging in such patients [42]. The authors of two recent reviews addressing clinical significance of lymphadenectomy in UTUC concluded that lymphadenectomy improves the staging and serves a therapeutic role in UTUC [43,44]. Some authors consider the absolute number of dissected lymph nodes (>6 or >8) an independent predictor of cancer-specific survival (CSS), while others indicate the role of the anatomic lymphatic templates as being more important than the extent of lymphadenectomy or the number of excised lymph nodes [25]. Our study showed that assessment of lymphatic outflow in cases of UTUC is possible, yet challenging. In our group of 19 patients, only two cases (10.5%) presented typical lymphatic outflow from the upper urinary tract with visualization of SLNs. Interestingly, in cases of tumors localized in the distal part of the ureter, SLN was seen outside the region of proposed lymphadenectomy for UTUC and would be missed during this procedure. In both cases where SLNs were visualized and the rest of the patients where UTUC was confirmed after biopsy, lymphadenectomy was not performed during the operation. Our study protocol did not include lymphadenectomy procedure according to the rules of the European Urological Association. This may be one of the goals of future studies. In five cases (26.3%), spillage of the radiotracer outside the urinary tract with probable gravitational leakage through the retroperitoneal space was noted. We suspect that in those cases the injection of the radiotracer could have been too deep, even though the length of the needle was adjusted to 2 mm before injection. Due to the thin wall of the ureter, it could have been punctured during injection without being noticed. In 12 cases (63.1%) the imaging revealed no lymphatic outflow from the injection site. We think that this could be due to a locally advanced tumor with possible metastatic cells blocking the outflow of the radiotracer through the lymphatic vessels. High pressure in the urinary tract during ureterorenoscopy could also obstruct the lymphatic vessels. In both cases where SLNs were visible, we have used standard intra-operative pressure ranging within 30–35 cmH_2_O. In the group of patients where SLNs were not visualized, the pressure had to be elevated above the standard 40 cmH_2_O for a short period of time for better visualization because of poor vision during ureterorenoscopy. We assume that this also could have been the reason for the lack of lymphatic outflow in the presented cases. Localization of the tumors where lymphatic outflow was observed was different. In both cases we observed a richly villous tumor on a wide peduncle. No specific features of the tumor were observed in either case. We can debate whether the proximity of the bladder wall in the case where the tumor was localized in the ureteral orifice could have played a role in lymphatic outflow, and the lymphatic system of the kidney in the case of the tumor localized in the renal pelvis. More studies are necessary to prove that. In the future, we plan to include patients with smaller tumors and use lower pressure during the ureterorenoscopy procedure. 

In this study we did not observe the crossover phenomenon. This finding is different from our previous study concerning urinary bladder cancer, where we observed this phenomenon in 45% of the cases in which lymphatic outflow occurred [34]. In the present study we found one single and one multiple (double), unilateral SLN, although this observation should be verified in larger cohorts of patients. 

A study of a different upper urinary tract tumor—kidney cancer—showed that the Sentinel Lymph Node Biopsy (SLNB) technique performed in this anatomical region using a radionuclide-based technique can clarify the pattern of lymphatic outflow and have diagnostic and therapeutic implications [24]. 

Diagnostic ureterorenoscopy with tumor biopsy in cases of suspicion of UTUC is in itself demanding, and performing additional procedures such as injection of radioisotope may sometimes be unsuccessful. 

## 5. Conclusions

Lymphatic outflow from the upper urinary tract is insufficiently studied. To our best knowledge this is the first study dealing with the subject of lymphatic outflow assessment and SLN identification in UTUC with the use of a radioisotope-based technique. Lymphatic outflow in this group of tumors is possible yet challenging, and SLNs can be visualized in about one out of ten patients by injection of radioisotopes during ureterorenoscopy with a tumor biopsy. In our studied group, both single and multiple SLNs were observed regarding the site of injection. Moreover, SLNs were localized outside the region of interest for the lymphadenectomy procedure in the case of UTUC. SPECT/CT provides information about unique patient anatomy of the lymphatic system and localization of LNs with the greatest chance of tumor spread. That information may potentially influence surgical treatment while deciding to perform lymphadenectomy and individualize treatments for patients.

The assessment of lymphatic outflow in UTUC using a radioisotope-based technique is possible yet challenging, with visualization of unilateral sentinel lymph nodes in about 10% of patients. The results of this procedure may potentially influence the extent of lymphadenectomies and individualize patient treatment.

## Figures and Tables

**Table 1:** Patients and tumor characteristics.

Patients n (%)	
Gender	
Male	7 (36.7%)
Female	12 (63.3%)
Age	
Median ≤ 69 years	61.6
Median > 69 years	79.4
Pathological stage	
pT0	8 (42%)
pTa	7 (36%)
pT1	4 (21%)
CIS	0
2004/2016 WHO grade	
Low grade	7 (36%)
High grade	4 (21%)
Lymphatic outflow analysis	
Number of patients with identified SLNs	2 (10.5%)
Number of patients with no lymphatic outflow	17 (89.5%)
Number of identified SLNs	2 (10.5%)

## Data Availability

Data supporting reported results can be found in Nuclear Medicine and Urology dataset of Medical University of Gdansk.
